# ED50 of ciprofol combined with different doses of remifentanil during upper gastrointestinal endoscopy in school-aged children: a prospective dose-finding study using an up-and-down sequential allocation method

**DOI:** 10.3389/fphar.2024.1386129

**Published:** 2024-10-11

**Authors:** Xu Zhang, Ning Zhang, Haicheng Song, Yueyi Ren

**Affiliations:** ^1^ Department of Anesthesiology, Peking University People’s Hospital, Qingdao, China; ^2^ Women’s and Children’s Hospital, Qingdao University, Qingdao, Shandong, China; ^3^ Department of Laboratory Medicine, Qingdao Women’s and Children’s Hospital Affiliated to Qingdao University, Qingdao, Shandong, China; ^4^ Department of Endoscopy Center, Qingdao Women’s and Children’s Hospital Affiliated to Qingdao University, Qingdao, Shandong, China

**Keywords:** children, ciprofol, median effective dose, remifentanil, upper gastrointestinal endoscopy

## Abstract

**Objective:**

This study aimed to determine the 50% effective dose (ED50) of ciprofol when combined with different doses of remifentanil for upper gastrointestinal endoscopy of school-age children and to evaluate its safety.

**Methods:**

This study involved school-aged children who were scheduled to undergo upper gastrointestinal endoscopy under deep sedation. The children were randomly assigned to two groups: remifentanil 0.3 μg/kg (R0.3) and remifentanil 0.5 μg/kg (R0.5). Anesthesia was induced with remifentanil, followed by ciprofol. The dose of ciprofol for each patient was determined using the Dixon up-down sequential method. If the MOAA/S score was ≤1 and the child did not exhibit significant movement or coughing during the endoscopy process, sedation was considered successful. The first patient in each group received 0.5 mg/kg ciprofol. The dose of ciprofol was adjusted by 0.05 mg/kg based on the response of the previous patient. The primary outcome was the ED50 of the ciprofol-induction dose. The total ciprofol doses, onset times, awakening times, and adverse reactions were recorded.

**Results:**

1) The Dixon method was used to collect crossovers data from each group, and the ED50 values of the R0.3 and R0.5 groups were calculated to be 0.390 mg/kg (95% CI 0.356–0.424 mg/kg) and 0.332 mg/kg (95% CI 0.291–0.374 mg/kg), respectively. The ED50 of ciprofol in the R0.3 group was significantly higher than that in the R0.5 group (*p* < 0.05). 2) The onset time and recovery time of the R0.5 group were shorter than those of the R0.3 group (*p* < 0.05). When the two groups were compared, the total dose of ciprofol in the R0.5 group decreased (*p* < 0.05). 3) Compared with the R0.3 group, the incidence of respiratory depression during induction in the R0.5 group increased (*p* < 0.05).

**Conclusion:**

This study explored the ED50 of ciprofol combined with different doses of remifentanil for successful sedation in upper gastrointestinal examinations in school-aged children. Compared to the use of remifentanil 0.3 μg/kg, the combination of ciprofol with remifentanil 0.5 μg/kg significantly reduced the ED50 required to prevent body movement or cough during endoscope insertion but increased the incidence of respiratory depression.

## Introduction

Pediatric patients typically require deep sedation or general anesthesia to successfully undergo upper gastrointestinal endoscopy without experiencing discomfort or traumatic memories (Chung and Lightdale, 2016). Propofol is widely used in upper gastrointestinal endoscopy because of its minimal residual effects and rapid onset and recovery (Kim et al., 2019). However, the use of propofol alone can lead to dose-dependent side effects, including respiratory depression, hypotension, and injection pain (Uliana et al., 2020). In particular, in pediatric patients, the injection of the pain agent propofol is a difficult issue. Even with the use of multiple methods, the incidence of propofol injection pain in children is still as high as 20% (Yan et al., 2015; Singla and Malde, 2018).

Ciprofol is a new type of intravenous anesthetic drug whose affinity for gamma-aminobutyric acid-A (GABAA) receptors is enhanced by the introduction of ciprofol groups into the chemical structure of propofol. The sedative effect of ciprofol is similar to that of propofol, but the incidence of injection pain and respiratory depression is significantly lower (Li et al., 2022; Chen et al., 2023). Two recent meta-analyses, grounded on randomized controlled trials, indicate that compared to propofol, ciprofol is a better alternative sedative for operations because its facilitates achieving a satisfactory anesthesia depth and results in fewer hypotension and injection-site pain (Akhtar et al., 2024; Ainiwaer and Jiang, 2024). Remifentanil is a potent opioid drug with rapid onset (approximately 1 min) and a shorter elimination half-life (10 min) (Ziesenitz et al., 2018). Compared with fentanyl, remifentanil significantly reduced the propofol dose, anesthesia onset time, eye opening time, and extubation time when propofol combined with opioids was used (Sridharan and Sivaramakrishnan, 2019). According to previous studies on upper gastrointestinal endoscopy in children, compared to the use of propofol alone, the use of propofol combined with 0.2–0.3 μg/kg remifentanil was effective at improving the quality of sedation, with reduced awakening time and propofol dosage (Uliana, G. N. et al., 2020). Compared with the combination of propofol and 1 μg/kg fentanyl, the combination of 0.5 μg/kg remifentanil significantly shortened the awakening time and reduced the dosage of propofol but was associated with more respiratory depression during induction (Hirsh et al., 2010).

Currently, ciprofol has been applied in clinical practice for the pediatric population. Recently, a clinical trial has confirmed that a combination of 0.6 mg/kg ciprofol and a low dose of rocuronium can provide satisfactory tracheal intubation conditions and ensure stable circulation and BIS in children undergoing daytime adenotonsillectomy (Pei et al., 2023). For the cardiac anesthesia of children with mild lesion congenital heart disease, ciprofol did not differ from propofol in terms of its effects on myocardial function and postoperative out-comes in children (Qin et al., 2024). The use of ciprofol in pediatric patients significantly reduced the incidence of injection pain and maintained good circulation stability. But there is a limited amount of research regarding the effectiveness and safety of combining ciprofol with opioids during upper gastrointestinal endoscopy procedures in pediatric patients. This trial aimed to determine the effect of remifentanil on the ED50 of ciprofol on blunting responses to gastroscopy in school children and to provide a reference for clinical application.

## Materials and methods

### Ethical approval

The study was approved by the Ethical Committee of Qingdao University Affiliated Women’s and Children’s Hospital (Qingdao, China) (No. QFELL-YJ-2023–64), and it was registered at Chinese Clinical Trials.gov (registration number: ChiCTR2300074880; date of registration: 18 August 2023). Written informed consent was obtained from the legal guardians of all the children.

### Patients

Patients aged 6–12 years who were scheduled for diagnostic upper gastrointestinal endoscopy under deep sedation/anesthesia without tracheal intubation between August 22 and 22 October 2023, were included (ASA Physical Status 1–2).

The exclusion criteria for patients were as follows: emergency endoscopic examination; upper respiratory tract infection; obesity (body mass index above 30 kg/m2); frequent vomiting; complex treatment required during examination; severe malnutrition; combined with other complex congenital diseases; and a history of anesthesia, sedation, or allergic reactions to the medication used in the past 7 days.

### Randomization and blinding

Patients were randomly assigned to two groups by using computer software with random numbers. Unblinded anesthesiologists who did not participate in patient care had access to the randomization code to allow for preparation of the study drugs and, in the case of an emergency event, during the procedure. Patients with emergency unblinding will be excluded from the study. The remifentanil (batch number: 081,106; Yichang Humanwell Pharmaceutical Company, Co., Ltd.) dose was drawn into a 10-mL syringe with normal saline added to produce a final volume of 10 mL of solution. The appropriate dose of ciprofol (batch number: H20200013, Liaoning Haisike Pharmaceutical Co., Ltd.) for that patient, as determined by the study protocol, was drawn into either a 10 mL syringe (subjects aged 8 years) or a 20 mL syringe (subjects aged >8 years) with normal saline, as per the product monograph, added to achieve final volumes of 10 and 20 mL, respectively. Participants and their parents, the anesthesiologist, the endoscopist, and the procedure room and recovery room nurses were blinded to patient assignment.

### Enrollment

Potential participants will be screened by an independent researcher the day before the surgery. The study protocol, potential risks, potential benefits, and alternatives will be explained to the children and their parents. In addition, parents will be informed that the data will be analyzed anonymously and participation in the study will not receive any payment. The independent researcher will be responsible for obtaining written informed consent and collecting demographic and baseline data from the participants.

### Anesthesia

A standardized anesthetic regimen was used. All patients were hospitalized and fasted for up to 6 h (for solids) or up to 2 h (for liquids) before endoscopic examination. Approximately 10 min before anesthesia in the waiting room, the patients were orally administered 6 mL of dyclonine 1% mucilage (manufactured by Yangtze River Pharmaceutical Group, China) for local anesthesia. Upon arriving at the operative area, children will be monitored with continuous electrocardiogram (ECG), heart rate (HR), respiratory rate (RR), pulse oxygen saturation (SpO2), and non-invasive blood pressure (NIBP). Those values will be recorded every 5 min. The patient was in a lateral position and inhaled oxygen through a heated and humidified nasal catheter (with an oxygen flow rate of 10 L/min and FiO_2_ of 60%). Vital signs were monitored continuously during the procedure.

Based on the preliminary experimental results, the initial dose of ciprofol used in the first case in both groups was 0.5 mg/kg. The dosage of ciprofol used by subsequent enrolled children was determined based on whether the previously enrolled child’s ciprofol dosage was successful. If the sedation was successful, the dosage of ciprofol used in the next patient was decreased by one dose. Conversely, if sedation is unsuccessful, the dosage of ciprofol used in the next patient will increase by one dose. The dose gradient of one ciprofol was defined as 0.05 mg/kg. According to the randomization code, remifentanil was administered intravenously at 0.3 μg/kg and 0.5 μg/kg. Due to its respiratory inhibitory effect, Remifentanil should be administered slowly through intravenous injection, with a rate of 1 mL every 3 s using a 10 mL syringe. Thirty seconds after remifentanil was given, the assigned dose of ciprofol was injected within 30 s. After administration, the sedation level of the patient was evaluated every 5 s. When the patient reached ≤1 on the Modified Observer’s Awareness/Sedation Assessment (MOAA/S) scale, an endoscope was immediately inserted. If the patient did not reach the target sedation depth within 1 min or experienced physical movement reactions during endoscopic insertion, an additional 0.1 mg/kg of ciprofol was administered until the examination was completed. The selection of the dose interval was based on 1) our own clinical experience and 2) the ease of calculating the dosage of ciprofol.

### Outcomes and definitions

The sedation status and MOAA/S scores of all pediatric patients during anesthesia induction will be assessed and recorded by another anesthesiologist. The primary outcome of this study was the ED50 of the ciprofol induction dose for successful sedation, and the secondary outcomes were the onset time, recovery time, cumulative dose of ciprofol and incidence of adverse reactions. Vital signs (MAP, HR, SpO_2_, RR) were recorded at different time points, including before anesthesia induction (T0/baseline), immediately after remifentanil administration (T1), immediately after ciprofol administration (T2), immediately after gastroscopy insertion (T3), and at the end of the examination (T4). The following adverse events were monitored during the surgery. If SpO_2_ < 95% (>15 s) occurred during the examination, it was defined as hypoxemia, and jaw thrust and increased oxygen flow were provided. An RR < 10 breaths/min was considered respiratory depression and was closely monitored. An RR < 5 breaths/min was considered respiratory arrest, and jaw stimulation was considered respiratory. If SpO_2_ was <90% at the same time, mask pressure oxygen was provided. A MAP decrease of more than 20% from the baseline was considered hypotension, and fluid replacement or vasoactive drugs were used to increase blood pressure. An HR < 60 times/min was considered to indicate bradycardia, and 0.1–0.3 mg of atropine was intravenously injected according to the patient’s condition. The occurrence of adverse reactions was recorded. Upon entering the recovery room, the MOAA/S score was evaluated every 5 min, and if the patient woke naturally, the MOAA/S score was immediately evaluated. The onset time was defined as the time from the completion of ciprofol administration to a MOAA/S score ≤1. The recovery time was defined as the time from the end of the examination to a MOAA/S score of ≥5.

### Data monitoring

No plans for intermediate analysis of the primary endpoint, but an independent Data and Safety Monitoring Board (DSMB) will regularly review the accumulated data and unblinded safety data of the study.

### Safety evaluation

Safety assessments will be composed of monitoring vital signs during the study and observing and recording all adverse events (AEs) and serious adverse events (SAEs). AEs, defined as all unfavorable/unexpected medical events that occur in patients, whether causally related to the study drugs or not, will be recorded and treated immediately. SAEs are defined asadverse medical events, such as death, life-threatening, permanent, or serious disability or loss of function, and prolonged hospitalization after the subject receives the investigational drug. SAEs will be reported to the Ethics Committee within 24 h. In addition, the researchers will purchase clinical trial insurance, which compensates for treating any harm that occurs during the study.

### Study discontinuation criteria

This trial will be terminated under the following criteria: 1) clustered serious adverse events are related to intervention measurement with supportive evidence and 2) the administration, including the DSMB, requests that the trial be discontinued.

### Risks and benefits

There are no additional risks in this study other than the potential risks of standard clinical practice. No participants will receive any direct benefits from the study nor any compensation for their participation.

### Sample size

This study used an up-and-down sequential method to calculate the ED50 of ciprofol combined with remifentanil. According to the research design requirements of the sequential method, at least 7 crossovers points with positive-negative reactions should be completed. Previous studies have focused mainly on the optimal dose of anesthetics under a sequential allocation design (BCD), and at least 20 to 40 patients are needed to provide stable target dose estimates for the most realistic situation (Görges et al., 2017; Oron al., 2022). To improve the authenticity and reliability of the experimental results, the sample size was appropriately increased (Chen et al., 2022). More crossovers points can lead to more accurate research results. Therefore, we designed a sample size of 40 cases per group and ended this study after obtaining 10 crossover points. A crossover point was defined as an unsuccessful sedation case turning into a successful sedation case. “Successful sedation” was defined if the MOAA/S score was ≤1 and the child did not exhibit significant movement or coughing that affected the examination procedure during endoscopic placement. Conversely, if within 2 min, the patient’s MOAA/S score was >1 or had a reaction to endoscopic placement, it was defined as “unsuccessful sedation”.

### Statistical analysis

All the statistical analyses were conducted using SPSS software (version 26.0). The Shapiro‒Wilk test was used to evaluate the normality of the data. Continuous normally distributed variables are represented by the mean ± standard deviation, and independent samples t tests were used for comparisons between groups. Nonnormally distributed data are represented by the median (quartile range) and were compared using the Wilcoxon rank sum test. The classified data are represented by n (%) and were analyzed using Fisher’s exact test. Dixon-Massey calculated the ED50 by taking the midpoint average of ineffective-effective crossovers. The ED50 (95% CI) of the ciprofol was calculated as the average of the midpoints of ineffective-effective crossovers and analyzed by an independent samples t-test for comparisons between groups. Probit regression calculation is a parametric regression that analyzes the statistical quantities of “effective” and “ineffective” responses for each dose category. Probit regression analysis is often used as a backup and sensitivity analysis to create dose-response curves. The Probit regression analysis was used to calculate the ED95 and 95% confidence interval (CI) of the ciprofol. Repeated measures analysis of variance was used to analyze the changes in vital signs over time between the two groups, with group as the intergroup factor and time as the intragroup factor. A significance level of *p* < 0.05 was considered to indicate statistical significance.

## Results

**FIGURE 1:**
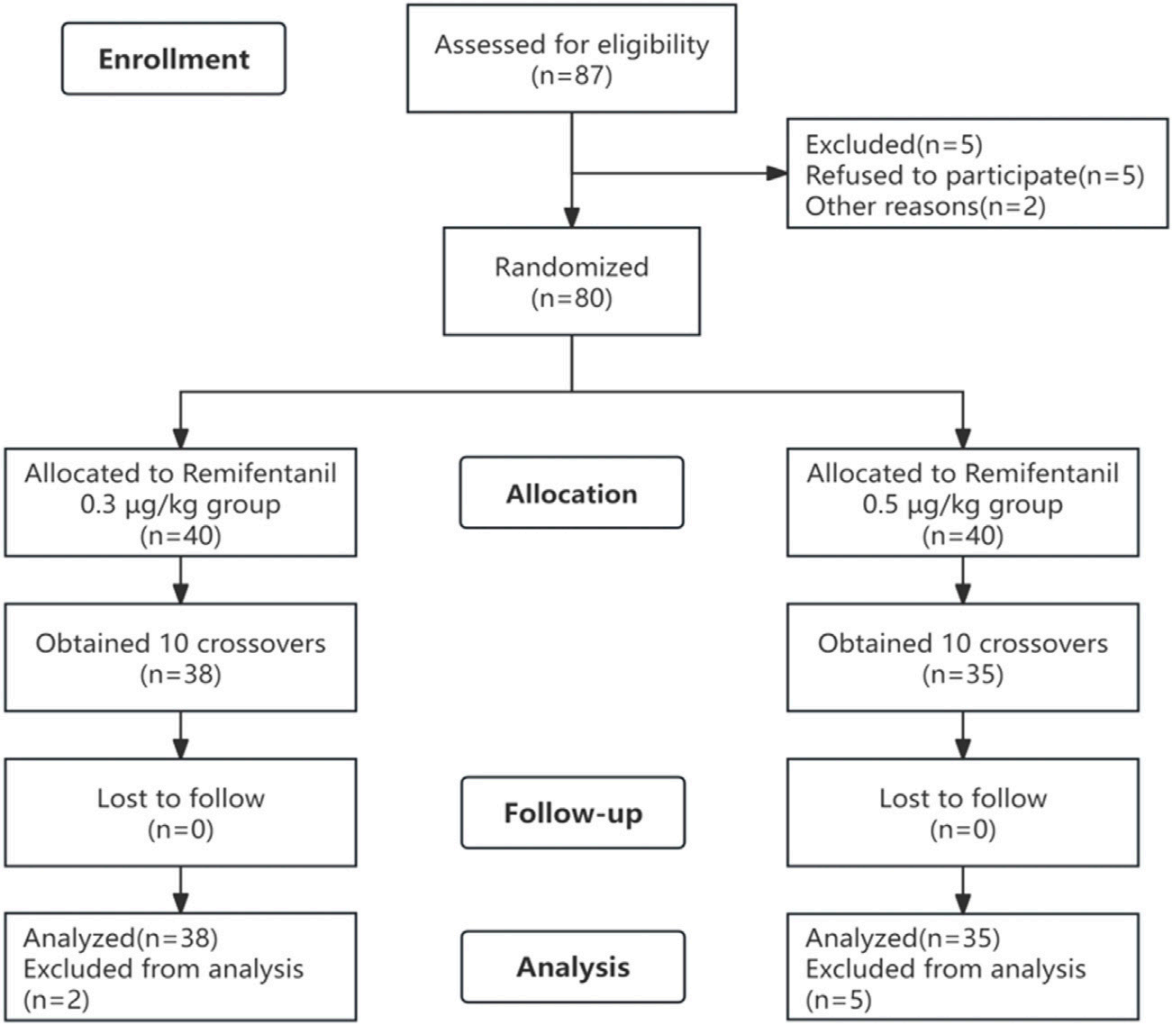
Flow chart of patients.

A total of 87 patients were initially screened. A total of 14 patients were excluded, including those who refused to participate (n = 5) or who experienced nausea and vomiting before the examination (n = 2), and the remaining patients (n = 7) were excluded after completing 10 crossovers (Figure 1). Finally, 38 patients in the R0.3 group and 35 patients in the R0.5 group participated in the study. There were no significant differences in the baseline characteristics (age, sex, BMI, or BSA) between the two groups (Table 1).

**TABLE 1:** Patient characteristics

Item	R0.3(Remifentanil 0.3 μg/kg) (n = 38)	R0.5(Remifentanil 0.5 μg/kg) (n = 35)	*P*
Age (years)	9.74 ± 1.93	9.37 ± 1.97	0.426^a^
Gender (Male/Female)	17/21	20/15	0.290^b^
BMI (kg/m^2)^	17.91 ± 3.66	18.52 ± 2.86	0.433^a^
BSA (m^2)^	1.33 ± 0.23	1.35 ± 0.23	0.713^a^

All values in the table represent the number or mean ± standard deviation.

BMI, body mass index; BSA, body surface area.

^a^ Indicates that the *p*-value is derived from the independent samples t-test.

^b^ Indicates that the *p*-value is derived from Pearson’s chi-squared test or Fisher’s test.

### Primary outcome

**FIGURE 2:**
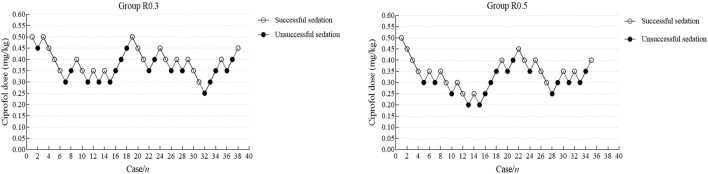
Individual response to upper gastrointestinal endoscopy in school-age children using a combination of cyclosporine and remifentanil at a corresponding dose (mg). Dixon-Massey up-and-down method for the group R0.3 **(A)** and the group R0.5 **(B)**.

**FIGURE 3:**
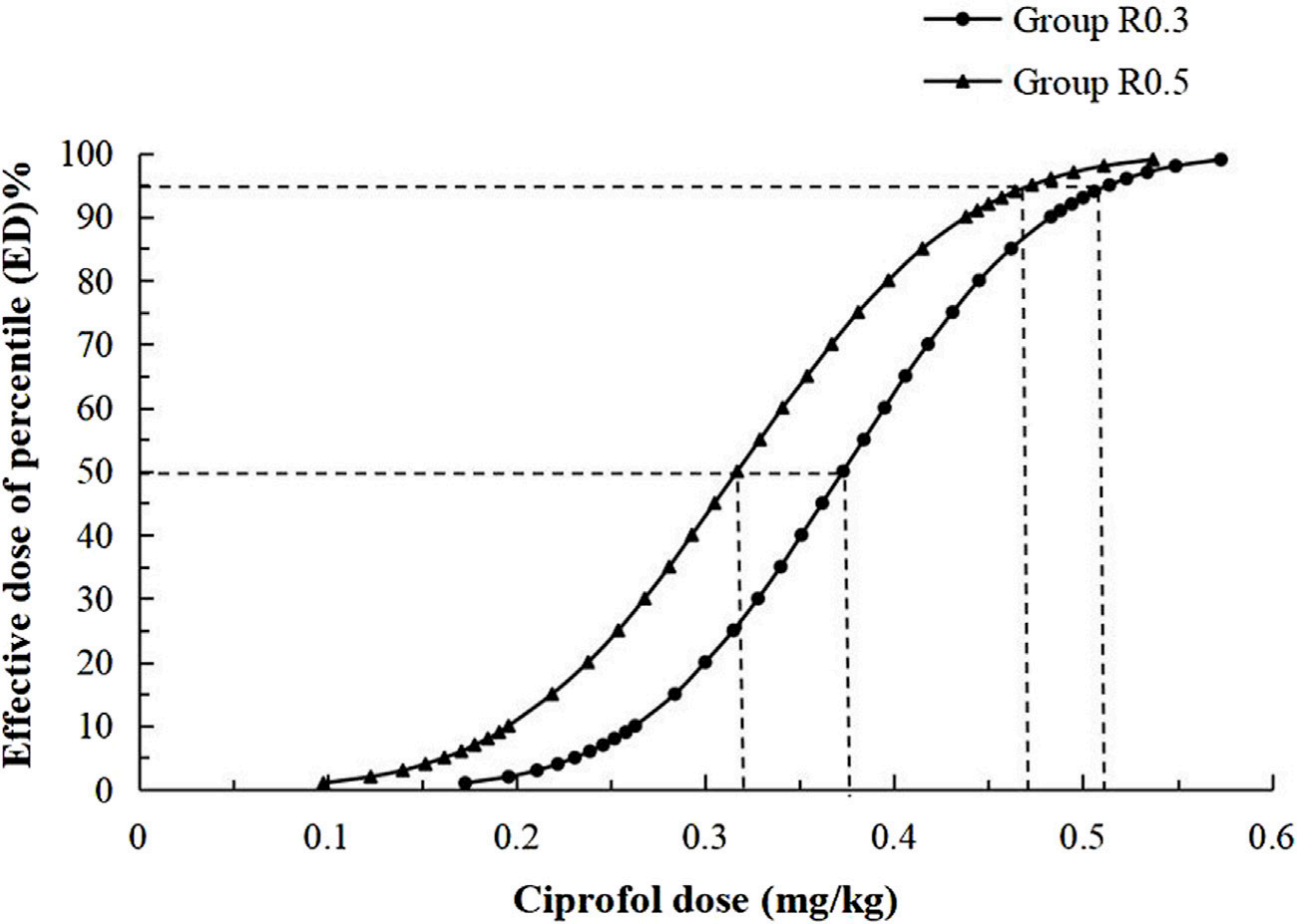
Dose-response curves of two groups for ciprofol derived from probit analysis. Dashed line indicates the position of the estimate of ED50 and ED95.

In this study, 10 crossovers were identified for each group, and the upper and lower sequences displaying the patient dose and response are shown in Figure 2. The Dixon method was used to collect cross-point data from each group, and the ED50 of the R0.3 group was calculated to be 0.390 mg/kg (95% CI 0.356–0.424 mg/kg). The ED50 of ciprofol in the R0.5 group was 0.332 mg/kg (95% CI 0.291–0.374 mg/kg). The ED50 of ciprofol in the R0.3 group was significantly higher than that in the R0.5 group (*p* < 0.05). According to the probit regression, the ED50 of ciprofol was 0.373 mg/kg (95% CI 0.316–0.421 mg/kg) in Group R0.3 and 0.317 mg/kg (95% CI 0.242–0.374 mg/kg) in Group R0.5. The ED95 values of the two groups were 0.514 mg/kg (95% CI 0.450–0.849 mg/kg) and 0.473 mg/kg (95% CI 0.401–0.929 mg/kg), respectively (Table 2). The dose–response curves for the ED50 and ED95 of ciprofol in the two groups are shown in Figure 3.

**TABLE 2:** ED50 and ED95 of ciprofol (with 95% confidence intervals) in the two groups.

	R_0.3_ (Remifentanil 0.3 μg/kg) (n = 38)	R_0.5_ (Remifentanil 0.5 μg/kg) (n = 35)	*P*
ED50 (Dixon–Massey) (mg/kg)	0.390 (0.356–0.424)	0.332 (0.291–0.374)	0.026^a^
ED50 (Probit regression) (mg/kg)	0.373 (0.316–0.421)	0.317 (0.242–0.374)	—
ED95 (Probit regression) (mg/kg)	0.514 (0.450–0.849)	0.473 (0.401–0.929)	—

A value of *P*< 0.05 was considered to indicate statistical significance between two groups.

^a^ Indicates that the *p*-value is derived from the independent samples t-test.

## Secondary outcomes

### Intraoperative and recovery indexes

There was no significant difference in procedure time between the two groups, but the onset time and recovery time of the R0.5 group were shorter than those of the R0.3 group (*p* < 0.05). When the two groups were compared, the total dose of ciprofol in the R0.5 group decreased (*p* < 0.05).

### Comparison of adverse events between the two groups

In the R0.3 group, 8 children (21.05%) developed hypotension, 5 children (13.16%) developed bradycardia, and 3 children (7.89%) developed respiratory depression. In the R0.5 group, 8 patients (22.86%) experienced hypotension, 8 patients (22.86%) experienced bradycardia, 9 patients (25.71%) experienced respiratory depression, and 3 patients (8.6%) experienced transient apnea. The incidence of respiratory depression in the R0.5 group was significantly higher than that in the R0.3 group (*p* < 0.05). There were 2 patients with myoclonus in each group. Neither group of children experienced injection pain or chest wall stiffness (Table 3).

**TABLE 3:** Examination-related characteristics and adverse reactions.

Item	R_0.3_ (Remifentanil 0.3 μg/kg) (n = 38)	R_0.5_ (Remifentanil 0.5 μg/kg) (n = 35)	*P*
Ciprofol dosage (mg)	20.82 ± 5.43	18.38 ± 4.27	0.037^a^
Onset time (s)	32.11 ± 7.77	23.86 ± 9.06	<0.001^a^
Examination time (min)	5.55 ± 0.86	5.20 ± 0.80	0.074^a^
Awakening time (min)	9.97 ± 3.30	7.94 ± 3.56	0.014^a^
Hypotension	8 (21.05)	8 (22.86)	0.852^b^
Bradycardia	5 (13.16)	8 (22.86)	0.279^b^
Respiratory depression	3 (7.89)	9 (25.71)	0.040^b^
Myoclonus	2 (5.26)	2 (5.71)	>0.999^b^
Dizzy	4 (10.53)	3 (8.57)	>0.999^b^

Values are presented as the mean ± standard deviation or the number of patients (%).

^a^ Indicates that the *p*-value is derived from the independent samples t-test.

^b^ Indicates that the *p*-value is derived from Pearson’s chi-squared test or Fisher’s test.

### Comparison of repeated measurements of vital signs between the two groups at different time points

**FIGURE 4:**
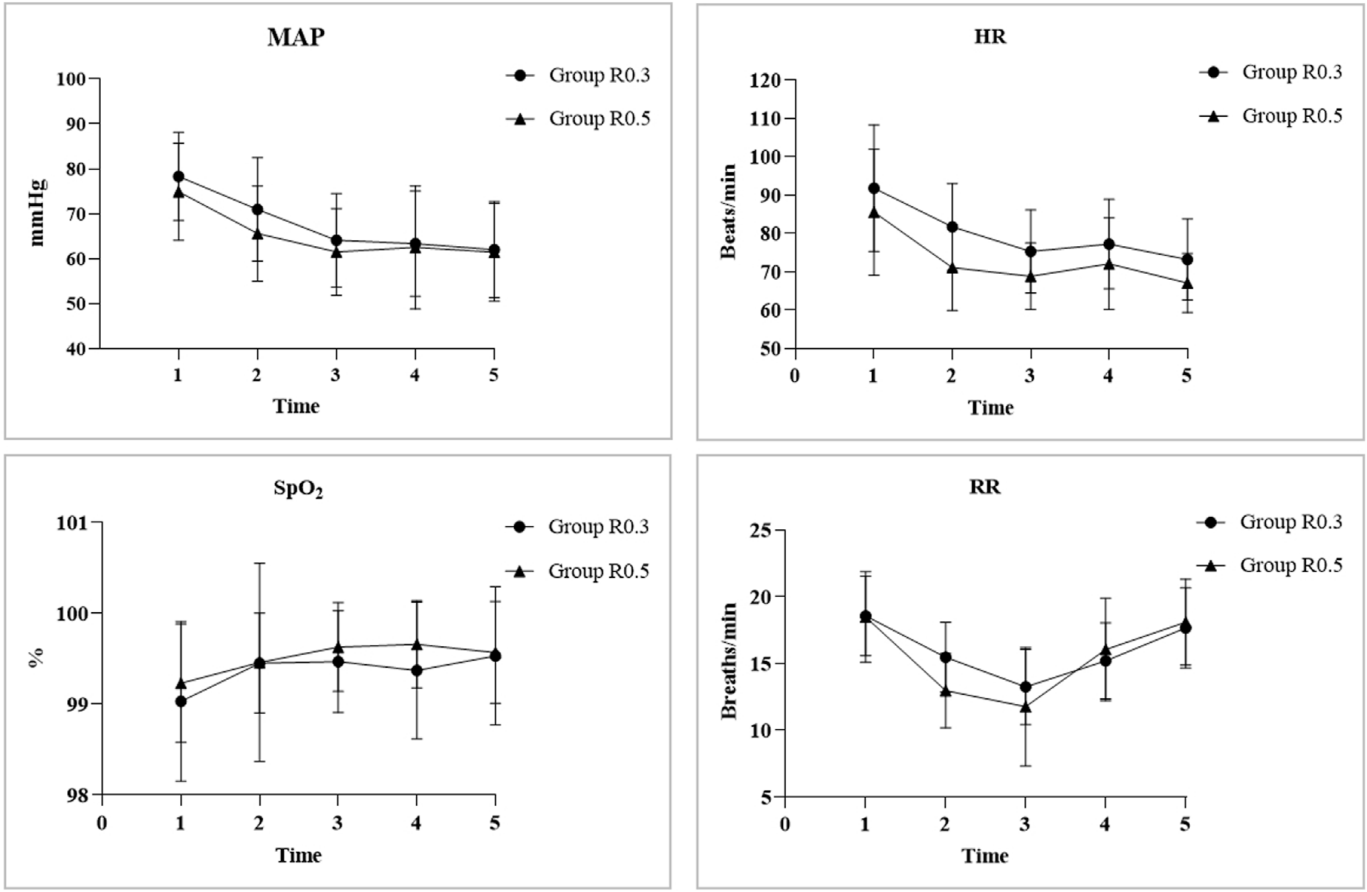
The MAP and HR of both groups showed a similar downward trend, with MAP and HR at T1, T2, T3, and T4 significantly lower than the baseline level of TO in the same group (all p < 0.05). Compared with TO, the RR of group R0.5 significantly decreased at T1, T2, T3, and T4. However, only group R0.3 showed a significant decrease in RR at T1, T2, and T3. Compared with TO, there was no significant difference in SPO2 between the two groups at each time point. There was no statistically significant difference in baseline indicators between the two groups. At T1, the MAP, HR, and RR of the group R0.5 were significantly lower than those of the group R0.3 (p < 0.05). “*” represents the time point had significant difference with TO (p < 0.05). “#” represents significant differences between groups (p < 0.05).

After induction, the MAP and HR of both groups showed a similar downward trend, with the MAP and HR at T1, T2, T3, and T4 being significantly lower than the baseline level at T0 in the same group (all *p* < 0.05). Compared with that at T0, the RR of group R0.5 significantly decreased at T1, T2, T3, and T4. However, only group R0.3 showed a significant decrease in RR at T1, T2, and T3. Compared with T0, there was no significant difference in SPO_2_ between the two groups at any time point. There was no statistically significant difference in the baseline indicators between the two groups. At T1, the MAP, HR, and RR of the R0.5 group were significantly lower than those of the R0.3 group (*p* < 0.05) (Figure 4).

## Discussion

In this study, the Dixon method was used to calculate that the ED50 of the R0.3 group was 0.390 mg/kg (95% CI 0.356–0.424 mg/kg), while the ED50 of the R0.5 group was 0.332 mg/kg (95% CI 0.291–0.374 mg/kg). We found that the use of 0.5 μg/kg remifentanil can reduce the ED50 of ciprofol for successful sedation in school-age children undergoing upper gastrointestinal endoscopy.

Intravenous anesthetics that act in different parts of the body typically exhibit synergistic effects, allowing each drug to be used at lower doses to enhance anesthesia and reduce side effects (Hendrickx et al., 2008). Propofol and remifentanil are known to have synergistic effects. Propofol is not an analgesic, and the addition of remifentanil reduces movement in response to surgical stimulation. During sedation during adult upper gastrointestinal endoscopy, propofol combined with low-dose remifentanil can effectively improve sedation quality, reduce awakening time, and improve recovery quality (Uliana et al., 2020). Propofol combined with remifentanil has good analgesic and sedative effects on pediatric minor surgeries, with a shorter recovery time and fewer delayed adverse reactions (Gul et al., 2014). Compared with the combination of ketamine and propofol, propofol combined with remifentanil can also be safely and effectively applied in pediatric endoscopy, providing a fast wake-up time and pleasant mood after waking, as well as fewer mental disorders and side effects (Damps et al., 2019). There is additivity of anesthetic effect when used remifentanil with propofol described in adults and in children 1 to 11 years, although effects such as apnea appear synergistic. A remifentanil infusion of 25 ng/kg/min reduced the concentration of propofol required for adequate anesthesia for esophagogastroduodenoscopy in children 3 to 10 years from 3.7 to 2.8 μg/mL (Fuentes et al., 2018). A pharmacodynamics model describing the propofol and remifentanil additive interaction for anesthesia in children 1 to 12 years using Bispectral Index as an effect measure is similar to that reported in adults (Anderson and Bagshaw, 2019). The mechanisms of action of ciprofol and propofol are similar, and compound opioid drugs are needed for clinical use (Teng et al., 2021). Compared with patients in the propofol-remifentanil group, patients in the ciprofol-remifentanil group had more stable hemodynamics. The lowest oxygen saturation in the ciprofol-remifentanil group was significantly higher than that in the propofol-remifentanil group (Wu et al., 2022). Therefore, the combination of ciprofol and remifentanil may be a good treatment option for sedation in children. In previous studies, the dose range of propofol combined with remifentanil for gastrointestinal endoscopy in children was 0.2–0.5 μg/kg. During the sedation process of the upper gastrointestinal tract, compared with the use of propofol alone, the combination of low doses of 0.2 μg/kg and 0.3 μg/kg remifentanil with propofol can significantly improve the quality of sedation, shorten the recovery time, and optimize the quality of recovery. Furthermore, no significant difference was observed in adverse events between the 0.3 μg/kg group and the 0.2 μg/kg group (Uliana, G. N. et al., 2020). Therefore, we did not include a placebo group of remifentanil but instead established a low-dose group of 0.3 μg/kg of remifentanil and a high-dose group of 0.5 μg/kg for the study.

The results of this study showed that after intravenous injection of remifentanil in both groups, the MAP, HR, and RR significantly decreased, while the SpO_2_ did not significantly change. On the one hand, most gastroscopic examinations are diagnostic, and the procedure time is short. To avoid a long recovery time and dizziness after examination, benzodiazepine sedatives were not used before the surgery. After entering the room, the child may be in a state of tension and anxiety, resulting in high baseline vital signs. On the other hand, the decrease in heart rate and blood pressure over time may be due to the synergistic effect of ciprofol and remifentanil on the cardiovascular system (Tirel et al., 2005). Comparing the two groups, at T1, the MAP, HR, and RR of the R0.5 group decreased more significantly. The analgesic and sedative effects of remifentanil are dose dependent. Higher doses of remifentanil can cause negative effects on the cardiovascular system and have a more significant slowing effect on the respiratory rate. At T2, the decrease in MAP was more significant in the R0.3 group because the R0.3 group received a higher dose of ciprofol, leading to a decrease in blood pressure.

Hypotension and bradycardia are common adverse reactions to anesthetics, propofol may reduce heart rate and blood pressure through brainstem autonomic control mediated by GABAA receptors. Compared to propofol, ciprofol has been shown to produce more stable hemodynamic responses during colonoscopy, but general anesthesia-induced hypotension and bradycardia still occur (Zhang et al., 2023; Lu et al., 2023). In adults, the incidences of hypotension and bradycardia in gastrointestinal endoscopy with the combination of propofol and fentanyl are 12.5% and 3.6%, respectively.^4^ After the administration of remifentanil, hypotension rarely occurs, but the negative impact of remifentanil on heart rate is significant, and a decrease in heart rate in children depends on variable susceptibility of the parasympathetic nervous system (Tirel et al., 2005). As in the endoscopic study of propofol combined with remifentanil, a statistically significant decrease in heart rate was observed in adults after administering remifentanil first (Uliana et al., 2020).

Although many sedatives have been shown to be safe and effective for endoscopic sedation, all have the potential to significantly depress the central nervous system, airway protective reflexes, and ventilation (Hartjes et al., 2021). As adverse reactions to remifentanil are mediated mainly by the activation of μ-opioid receptors and are related to the dosage and concentration of the effector site, respiratory inhibition is the most relevant adverse reaction (Ziesenitz et al., 2018). The respiratory inhibitory side effects of remifentanil most commonly occur during the induction process. Several studies have shown that, compared with fentanyl and sufentanil, remifentanil results in more respiratory suppression and apnea during anesthesia induction, but there is no apnea during or after surgery (Zhao et al., 2015). Hirsh et al. (2010) noted that when propofol was used in combination with 0.5 μg/kg of remifentanil, the incidence of apnea during gastroscopy in children was 31.8%, and the incidence of hypoxemia was 27.3%. Gul et al. (2014) revealed that when propofol was used in combination with 0.25 μg/kg of remifentanil, the incidence of apnea during gastroscopy in children was 43.8%. Notably, the average SpO2 value in the former remifentanil group was only 82.3%, while that in the latter group reached 97.1%. This difference may be due to multiple factors, including the dose of remifentanil used and the flow rate of inhaled oxygen during induction. In their studies, apnea was observed after induction in most patients, especially in the remifentanil group. However, this situation was not clinically significant since spontaneous breathing started in all patients, either by themselves or with a simple intervention. In our study, the R0.5 group patients had a higher incidence of respiratory depression (26%), and 3 (9%) patients experienced transient apnea. Apnea was so brief that ventilation recovered quickly after a gentle mandibular thrust. Although the incidence of respiratory depression in this study was lower than that in previous studies, it still needs attention. Combined with this study and previous research literature on remifentanil, we have some recommendations. 1)The use of low-dose remifentanil combined with cyclopofol can be a good way to complete the upper gastrointestinal examination of school-age children. 2) For children at high risk of respiratory depression, such as neurodevelopmental disorders, obstructive sleep apnea syndrome and suspected difficult airway, try to avoid using high-dose remifentanil (Joosten et al., 2017; Jay et al., 2017). 3) After injection of 0.5 μg/kg remifentanil in healthy volunteers, the slope of the carbon dioxide ventilation response curve decreased and shifted downward, reaching its nadir at about 2.5 min after injection (Babenco et al., 2000). 4) Hypoxemia was not present in this study, suggesting that the use of high-flow nasal oxygen may reduce the incidence of peripheral oxygen desaturation in patients at risk of hypoxemia when undergoing gastrointestinal endoscopy under deep sedation (Nay et al., 2021). Previous study have shown that high inspired oxygen concentration increases the speed of onset of remifentanil-induced respiratory depression (Dahan et al., 2016). Additional studies have demonstrated that nasal high-flow oxygen with 30% oxygen exhibits significantly shorter apnea times than with 100% oxygen mode (Riva et al., 2018). 5) The vast majority of sedation complications in pediatric upper gastrointestinal endoscopy can be managed with simple maneuvers, such as supplemental oxygen, opening the airway, suctioning, placement of an oral or nasopharyngeal airway, and bag-mask-valve ventilation. Rarely, tracheul intubation is required for more prolonged ventilatory support. 6) Of particular note a state of deep sedation may be accompanied by partial or complete loss of protective airway reflexes. Patients may pass from a state of deep sedation to the state of general anesthesia. The anesthesiologist must have capable of managing any airway, ventilatory, or cardiovascular emergency event resulting from the deep sedation and/or general anesthesia (Coté et al., 2019).

No nausea or vomiting occurred in either group of children. No injection pain occurred during the injection of ciprofol, and analysis suggested that the incidence of injection pain in the ciprofol itself was relatively low and that the combination of remifentanil further reduced the occurrence of injection pain (Yan et al., 2015).

Our study has several limitations. First, our study was conducted in a specialized hospital with endoscopists who have extensive clinical experience and are familiar with the examination procedures. Due to technical differences, the results of this study may differ for primary practitioners. For beginner endoscopists, the recommended dose of ciprofol combined with remifentanilcombined in our study cannot be extended to these populations. Second, it was mentioned in the discussion that all the children did not receive preoperative sedation, which may have exaggerated the inhibitory effect of dose combinations on the cardiovascular system. Finally, we designed only two dose studies, and for child protection reasons, we did not establish a blank dose group. Additionally, higher doses (remifentanil) may further reduce the ED50 of ciprofol, but patients face a high incidence of respiratory depression. Future research should include prospective cohort studies based on the ED95 of ciprofol obtained in this study to explore the efficacy and safety of ciprofol in combination with remifentanil.

In summary, this study explored the ED50 of ciprofol combined with different doses of remifentanil for successful sedation in upper gastrointestinal examinations in school-aged children. The combination of ciprofol with remifentanil 0.5 μg/kg significantly reduced the ED50 but increased the incidence of respiratory depression.

## Data Availability

The original contributions presented in the study are included in the article/supplementary material, further inquiries can be directed to the corresponding author.
